# Mitochondrial Dysfunction and Endoplasmic Reticulum Stress in Age Related Macular Degeneration, Role in Pathophysiology, and Possible New Therapeutic Strategies

**DOI:** 10.3390/antiox10081170

**Published:** 2021-07-23

**Authors:** Valentina Bilbao-Malavé, Jorge González-Zamora, Miriam de la Puente, Sergio Recalde, Patricia Fernandez-Robredo, María Hernandez, Alfredo Garcia Layana, Manuel Saenz de Viteri

**Affiliations:** 1 Department of Opthalmology, Clínica Universidad de Navarra, 31008 Pamplona, Spain; vbilbao@unav.es (V.B.-M.); jgzamora@unav.es (J.G.-Z.); mdelapuentec@unav.es (M.d.l.P.); aglayana@unav.es (A.G.L.); 2 Retinal Pathologies and New Therapies Group, Experimental Ophthalmology Laboratory, Department of Ophthalmology, Universidad de Navarra, 31008 Pamplona, Spain; srecalde@unav.es (S.R.); pfrobredo@unav.es (P.F.-R.); mahersan@unav.es (M.H.); 3 Navarra Institute for Health Research, IdiSNA, 31008 Pamplona, Spain; 4 Red Temática de Investigación Cooperativa en Salud: ‘Prevention, Early Detection, and Treatment of the Prevalent Degenerative and Chronic Ocular Pathology’ from (RD16/0008/0011), Ministerio de Ciencia, Innovación y Universidades, Instituto de Salud Carlos III, 28029 Madrid, Spain

**Keywords:** age related macular degeneration, mitochondria, endoplasmic reticulum, oxidative stress, antioxidants, bile acids, resveratrol, melatonin, humanin, coenzyme Q10

## Abstract

Age related macular degeneration (AMD) is the main cause of legal blindness in developed countries. It is a multifactorial disease in which a combination of genetic and environmental factors contributes to increased risk of developing this vision-incapacitating condition. Oxidative stress plays a central role in the pathophysiology of AMD and recent publications have highlighted the importance of mitochondrial dysfunction and endoplasmic reticulum stress in this disease. Although treatment with vascular endothelium growth factor inhibitors have decreased the risk of blindness in patients with the exudative form of AMD, the search for new therapeutic options continues to prevent the loss of photoreceptors and retinal pigment epithelium cells, characteristic of late stage AMD. In this review, we explain how mitochondrial dysfunction and endoplasmic reticulum stress participate in AMD pathogenesis. We also discuss a role of several antioxidants (bile acids, resveratrol, melatonin, humanin, and coenzyme Q10) in amelioration of AMD pathology.

## 1. Introduction

Age-related macular degeneration (AMD) is a progressive chronic disease of the central retina that affects older adults and is the major cause of blindness in developed countries [1,2,3]. AMD globally affects approximately 8.7% of individuals between 45 and 85 years of age, and 20–30% of people over 75 years in the developed world [4]. As a consequence of the increased aging of the population, it is expected that the number of patients affected by AMD will be close to 288 million by 2040, and the prevalence will increase by 15% in 2050 [4].

In early stages, AMD is characterized by drusen (small yellow deposits of lipids and proteins under the retina) and focal abnormalities of the retinal pigment epithelium (RPE). The RPE is a polarized monolayer of post-mitotic cells that sits between the photoreceptors and the choriocapillaris [5]. Photoreceptors are exposed to high levels of light and the retina produces many photo-oxidative radicals due to a high metabolic activity, which can affect the function and structure of RPE and other retinal cells. Among many functions, RPE is responsible for the elimination of metabolic end products from the photoreceptors and participates in the retinoid cycle. In addition, it transports glucose and other nutrients from the choroidal circulation to the photoreceptors [6,7].

**Figure 1:**
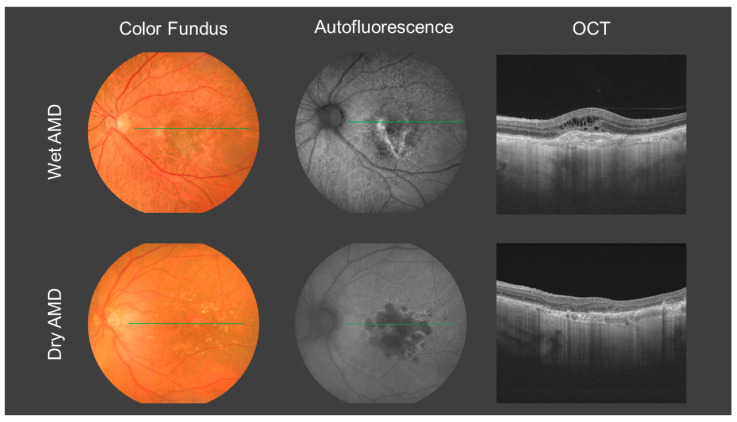
Representative retinographies and optical coherence tomography (OCT) of and eye with wet AMD and an eye with dry AMD. (**Top row**): Color fundus retinography, autofluorescence and OCT of the left eye of a patient presenting wet AMD. In retinography, choroidal neovascularization (CNV) can be seen as a grayish region covering the central macular area. Autofluorescece imaging shows a mixed pattern of hyperautofluorescence and hypoautofluorescence. On OCT the CNV can be clearly visualized as a hyperreflective layer under the retina with intraretinal fluid visualized as multiple hyporeflective spaces. (**Bottom row**): Color fundus retinography, autofluorescence, and OCT of the left eye of a patient presenting advanced dry AMD. In the retinography an area of central atrophy can be observed in the central macular area that allows better visualization of the choroidal vessels and is surrounded by multiple drusen. Autofluorescence clearly distinguishes the borders of the geographic atrophy due to hypoautofluorescence secondary to the retinal pigment epithelium (RPE) atrophy. OCT reveals atrophy of the RPE and outer retinal layers, with increased signal in the choroid and sclera. The green line represents the OCT section location.

In late stages, AMD can be characterized by two different phenotypes (Figure 1). In dry or non-neovascular AMD, geographic atrophy (GA) appears, with loss of the outer retina, RPE and choriocapillaris; whereas in wet or neovascular AMD, choroidal neovascular membranes (CNM) develop, leading to an important loss of vision when the fovea (the central part of the macula) is affected [1,2].

AMD is a multifactorial disease; its etiology comprehends several genetic and environmental factors. The most important risk factor is age, but there are others such as genetics, race, family history, smoking, hypertension, and low dietary intake of antioxidants (vitamins E, C, zinc, and carotenoids) [1,3,8]. AMD is a disorder with complex pathological changes that include dysregulation in the complement, lipid, angiogenic, inflammatory and extracellular matrix pathways, and RPE dysfunction plays a crucial role in its pathophysiology [1,9].

Currently, intravitreal administration of anti-vascular endothelial factor (VEGF) inhibitors is used to treat CNV in patients with wet AMD, while no preventive or curative therapy exists for dry AMD [2]. However, different antioxidant formulations and mineral supplementation have been studied and are widely used as they have shown to reduce the 5-year risk of progression from intermediate to advanced AMD by approximately 25% [10].

Given the importance of oxidative stress in the pathophysiology of AMD and the existing need to find more effective therapies to prevent blindness from this disease, we performed this review that explains two important processes implicated in cellular response to oxidative stress: mitochondrial and endoplasmic reticulum stress, as well as possible therapies that have been studied to mitigate their deleterious effects in patients with AMD.

## 2. Mitochondria and AMD

### 2.1. Retinal Pigment Epithelium and Mitochondria

Increasing knowledge is highlighting the importance of the RPE in the pathogenesis of AMD and oxidative stress is a key factor for triggering RPE degeneration [11]. Reactive oxygen species (ROS) are produced mainly in the mitochondria, a doubled membrane-bound organelle (Figure 1) present in almost all eukaryotic cells in a density that varies to match their metabolic demands. In the RPE, mitochondria are abundant and constitute the main source of energy. This organelle produces energy in the form of adenosine triphosphate (ATP) via oxidative phosphorylation (OP), a metabolic process that involves the transfer of electrons between the subunit complexes of the electron transport chain (ETC) coupled with the reduction of oxygen. However, up to 5% of the oxygen is incompletely reduced, leading to elevated ROS and superoxide production [11,12]. Mitochondria are highly dynamic organelles that, in addition to generating energy, perform other important functions essential for cell survival, including calcium buffering or the metabolism of cholesterol and iron [13]. Maintaining mitochondrial homeostasis is a delicate process achieved by fusion, fission, biogenesis and mitophagy, along with a coordinated expression of genes from nuclear and mitochondrial DNA (mtDNA) [13].

### 2.2. Mitochondria and Retinal Senescence

Mitochondrial dysfunction has been associated with normal aging, as well as a variety of age-related diseases [14]. Recent studies have confirmed that aged individuals have fewer and smaller mitochondria in the RPE [12]. Moreover, mitochondrial ROS and oxidative damage are significantly increased with aging [11]. Cellular senescence is an adaptative biological response to a variety of exogenous and endogenous stimuli that arrests cell replication. Despite its importance during development, tissue regeneration, and as a preventive mechanism against tumor proliferation, an accumulation of senescent cells is a driver of the aging process and it is implicated in pathogenesis of several age related diseases, including AMD [14,15,16]. Mitochondria play a key role in the regulation of the senescent phenotype. Although mitochondria are more abundant in senescent cells, their morphology and function are severely altered due to alterations in fusion and fission mechanisms, decreased membrane potential, increased proton leak, and ROS production [14]. This excessive production of mitochondrial ROS produces DNA damage, particularly at the telemore regions contributing to the induction of senescence [14].

In the human aging retina, RPE, ganglion, amacrine, and horizontal cells, and rods acquire characteristics of cellular senescence [16]. Age related accumulation of senescent RPE cells is marked around drusen, and amyloid-beta (a component of drusen) can induce RPE senescence [15,16]. Moreover, A2E a fluorophore present in lipofuscin (which accumulates in the aging RPE) can also induce cellular senescence by damaging DNA and shortening telomeres [17]. Senescence has also been found in retinal and choroidal vascular cells and the consequent vascular dysfunction is associated with the progression of AMD [16].

Another key factor of cellular senescence is the senescence-associated secretory phenotype (SASP), wherein senescent cells release growth factors, cytokines, chemokines, proteases, and other molecules that induce inflammation and other AMD-related effects [16,17]. Senescent RPE cells secrete higher levels of MMP-9 and IL-8 which can promote the breakdown of the blood–retinal barrier by increasing tight junction´s degradation and activation of immune cells [17].

It is well known that genetic variations in the complement factor H (CFH) and the age-related maculopathy susceptibility 2/Htra serin peptidase 1 (ARMS2) genes play an important role in the risk of developing AMD [18]. Oxidative stress has been shown to induce an abnormal transcription and production of HTRA1 in human RPE, and excessive expression of this protein can induce RPE senescence. Moreover, RPE senescence has been shown to downregulate the expression of CFH while upregulating VEGF expression [19].

### 2.3. Mitochondrial Dysfunction and AMD

The importance of mitochondrial dysfunction in the pathogenesis of AMD has granted the attention of researchers as evidence suggesting a decline in mitochondrial function in people with AMD is increasing. Data support a reduction in the number of mitochondria in AMD patients when compared to age-matched controls [13,15,20]. Patients with AMD also have pronounced damage to the mitochondrial structure with a partial or total loss of their cristae [20,21], confirmed by electron micrographs or the increase in the content of mitofilin (a protein involved in cristae stabilization) observed in patients with early AMD [13]. Although mitophagy, the mechanism for the removal of abnormal mitochondria, has not been directly measured, it is likely that this important mechanism is impaired, especially as evidence suggests that autophagy in the RPE decreases due to chronic exposure to mitochondrial oxidative stress [13,20,22].

Recent studies have focused on the metabolic codependence between the RPE and the retina, using the term “metabolic ecosystem” to describe it. While photoreceptors use glucose to generate ATP via glycosis, RPE relays mostly on the mitochondrial OP to generate the energy needed to meet their high metabolic demands [13,23]. Lactate produced by photoreceptors is transported to the RPE and might be the signal that inhibits RPE from using glucose [23]. However, in AMD, mitochondrial dysfunction forces the RPE to rely on glucose and glycolysis to supply its energy requirements breaking the “metabolic ecosystem” and leading to PR starvation and subsequence death [13,21].

### 2.4. Mitochondrial DNA Damage in AMD

Total mitochondrial DNA content decreases with age with possible consequences for ATP production in the RPE [24]. Furthermore, increased ROS levels promote oxidative damage to the mtDNA and normal aging is associated with accumulation of mtDNA mutations in RPE, choroid, photoreceptors and ganglion cells [15]. Compared to nuclear DNA, mtDNA is more susceptible to oxidative damage due to several factors: the lack of protective protein complexes such as histones, its location in close proximity to the ROS generating ETC, and fewer mechanisms to repair DNA damage [24]. Moreover, efficacy of the repair of mtDNA in the RPE is more reduced in the macula compared to the peripheral retina [15]. In a vicious cycle, damaged mtDNA induces mitochondrial dysfunction with disturbed OP and higher ROS production [25]. Interestingly, age related damage mtDNA is limited to only one region of the mitochondrial genome that encompasses the “common deletion” (CD), a 4977 pair deletion frequently seen in patients with mitochondrial diseases and in other aged postmitotic and quiescent cells like the RPE [15,24]. On the contrary, damage to the mtDNA in patients with AMD is distributed throughout the mitochondrial genome [24]. Furthermore, mtDNA damage corralates with AMD severity [15]. It is also important to note, that AMD related mtDNA damage has not observed in photoreceptors, probably related to the difference in methabolic pathways used by different retinal cells to generate energy [15]. Mitochondrial haplogroups are collections of similar haplotypes (defined by combinations of single nuclear polymorphisms in mtDNA) and some studies have shown that the risk for developing AMD varies in different mitochondrial haplogroups [25]. Moreover, case control studies have found that the presence of high risk allele for CFH is significantly associated with mtDNA damage [26] and the A69S SNP in ARMS2 may lead to mitochondrial dysfunction in patients with AMD [27].

## 3. Endoplasmic Reticulum and AMD

### 3.1. The Endoplasmic Reticulum Normal Functioning: Protein Folding

**Figure 2:**
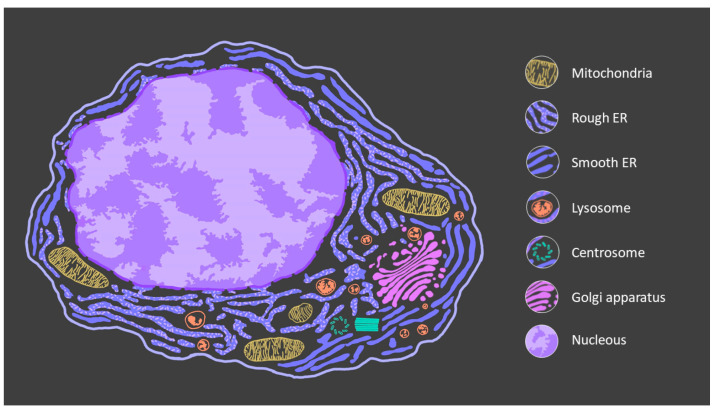
Gross architecture of a eukaryotic cell.

The endoplasmic reticulum is the largest organelle in cells made up of an extensively folded tubulovesicular membrane network matrix (Figure 2). It has several essential functions such as Ca^2+^ storage, lipid metabolism, and protein synthesis [28]. In fact, it is the principal site for synthesis of proteins, post-translational modification, folding, and assembly of newly synthesized protein [29].

Although protein synthesis is initiated in the cytoplasm, many of the synthesized proteins are destined to be expressed on the cell surface or to be secreted, and the endoplasmic reticulum is the organelle in charge of this processing. In eukaryotic cells, approximately one-third of proteins go through a series of modifications in the endoplasmic reticulum, in which they enter in a predominantly unfolded state and leave it to move through the Golgi apparatus and secretory vesicles in a folded state and, in cases of complexes composed of several subunits, assembled [30].

To achieve their native tertiary and quaternary structure, proteins must undergo a series of processes such as signal sequence cleavage, glycosylation, disulfide bonding, and pro-isomerization. These tasks are carried out by a great variety of chaperones and enzymes specialized in different aspects of the folding process [31]. The first steps in protein folding are catalyzed by peptidyl prolyl isomerases, and chaperones such as GRP/94 and BiP (also known as GRP78) are responsible for maintaining a competent folding state and avoiding unwanted interactions [32]. Glycosylation of glycoproteins involves glycosidases and mannosidases while lectin-like chaperones such as calnexin and calreticulin are responsible for glycoprotein quality control allowing misfolded proteins multiple chances to acquire a correctly folded conformation in de-glycosylation and re-glucosylation cycles (calnexin/calreticulin chaperone cycle) [33].

Exposure of hydrophobic residues, which can occur during any step of the folding process, results in unwanted interactions within or between different polypeptide chains, leading to protein misfolding and often aggregation [34,35]. Therefore, a critical function of ER chaperones is to avoid non-productive or inappropriate interactions, and consequently prevent protein misfolding and aggregation. As an example, BiP, one of the best characterized endoplasmic reticulum chaperones, interacts with many secretory and membrane proteins during their maturation and is thought to bind short sequences of non-polar, hydrophobic amino acids, which in mature proteins are usually clustered inside proteins avoiding contact with water molecules that envelop the protein surface [34]. Therefore, BiP will have a higher binding affinity to unfolded or misfolded proteins. Normally the interactions are weak and short-lived; however, a large fraction of proteins cannot fold correctly, as occurs when the internal environment of the endoplasmic reticulum is perturbed or the protein structure is altered as a result of mutation, and consequently unfolded proteins often form stable and prolonged binding interactions with BiP and are retained in the lumen of the ER [36]. This interaction targets the proteins for the refolding process. If refolding fails, misfolded proteins will be removed from the ER by the ER-associated degradation machinery (ERAD) to preserve ER homeostasis [37].

In general, misfolded proteins are removed promptly through the ERAD by targeting proteins to the proteasome, a large multi-catalytic protease that resides in the cytoplasm. However, when proteins generate aggregates or are too large, another pathway called ER-to-lysosomes-associated degradation (ERLAD) emerges and engulfs the ERAD-resistant proteins into vesicles and delivers the cargoes to the lysosome for degradation [38].

Upon completion of folding, mature proteins are released from ER chaperones and then are transported to the Golgi apparatus. The dysfunction of the ER causes the accumulation of unfolded or misfolded proteins, a condition usually termed as ER stress that triggers the so-called unfolded protein response (UPR) to restore ER proteostasis.

### 3.2. ER-Stress and Adaptive Mechanisms

Under certain circumstances, such as hypoxia, oxidative stress and inflammation, the protein-folding capacity of the reticulum can be impaired and lead to the build-up of unfolded proteins in the ER lumen. To overcome this situation, the cell displays adaptive mechanisms such as increased ERAD, reticulophagy, and UPR.

#### 3.2.1. ERAD

**Figure 3:**
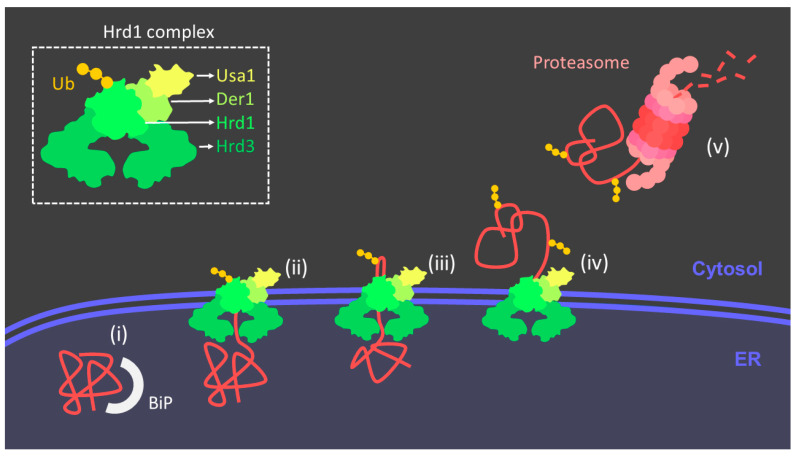
Recognition, retro-translocation, polyubiquitination, and proteasomal degradation of soluble ERAD Substrates. (**i**) Recognition of misfolded proteins by the ER-resident chaperones like BiP targeting them for retro-translocation. (**ii**) Initiation of retro-translocation crossing the Hrd1 complex, constituted by several subunits such as Hrd1, Hrd3, Der1, and Usa1; other subunits are not represented. (**iii**) The catalytic domain of Hrd1 polyubiquitinates the emerging protein in the cytosol. (**iv**) Further retro-translocation and polyubiquitination aided by cytoplasmic ubiquitin-binding protein complexes (not shown). (**v**) Proteasome degradation where the protein is broken down into peptide fragments.

As explained above, misfolded proteins can be eliminated by the proteasome in the so-called ERAD. This process follows a series of steps: protein recognition, protein targeting, retrotranslocation, ubiquitylation, and proteasomal degradation mainly [37] (Figure 3). First, the cell has to discriminate correctly folded proteins from misfolded ones, this protein recognition is thought to be not completely specific, and some correctly folded proteins end up being degraded in favor of ensuring that misfolded proteins do not accumulate as the cell cannot afford the risk of the acquiring toxic aggregates. As mentioned, in the native conformation, the hydrophobic chains of amino acids are usually buried inside soluble proteins avoiding contact with water molecules; however, these hydrophobic patches can be exposed in the unfolded state, which can lead to aggregation between the hydrophobic chains of other proteins. Cytoplasmic and luminal chaperones, such as 70 kDa heat-shock protein (Hsp70)-family members (e.g., BiP), calnexin and calreticulin, bind to these proteins in stable interactions retaining this proteins in the ER lumen [39]. When these proteins linger too long in the ER, they are susceptible to modifications that allow them to be recognized by other proteins that initiate retrotranslocation, this process is best known in glycosylated proteins [40]. In order for the proteins to be cleared they must be ubiquitinated and degraded by the proteasome, intriguingly, no enzymes associated with either process have been reported to exist in the ER lumen, therefore, a mechanism capable of controlling the transport of these proteins across the ER-membrane is indispensable [41]. The retrotranslocation takes place through a complex of proteins that is constitute by a RING-finger ubiquitin ligase Hrd1 and four additional proteins (Hrd3, Usa1, Der1, and Yos9) [42]. Hrd1 is activated by autoubiquitination on its cytosolic side [43]. Once in the cytosol, substrates are then polyubiquitinated by Hrd1p, which recruits ubiquitin-conjugating enzymes [44]. The ubiquitinated protein is pulled out the membrane by the cytosolic Cdc48 ATPase and its cofactor Ufd1/Npl4. The Cdc48 ATPase employs the energy of ATP hydrolysis to progressively retro-translocate the protein [45]. Ultimately, the protein is transported to the proteasome for degradation.

#### 3.2.2. Reticulophagy

**Figure 4:**
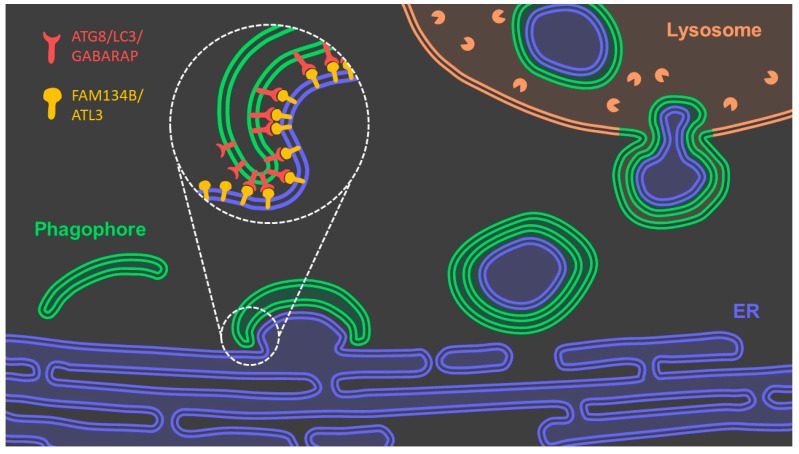
Schematic illustration of reticulophagy. Reticulophagy receptors FAM134B/ATL3 present in the surface of the ER-membrane are recognized by ATG8/LC3/GABARAP proteins in the surface of the phagophore. As a result of this interaction fragments of the ER are enclosed by the phagophore, forming an autophagosome, which delivers the enclosed ER fragments to the lysosome for degradation.

Together with the proteasome, macroautophagy is a main pathway for the degradation of intracellular elements [46]. Reticulophagy is the selective clearance and degradation of the ER components and membranes by encapsulation of fragments of the ER in forming autophagosomes (Figure 4). In order for specific fragments of the ER to be sequestered within autophagosomes there must be a recognition mechanism between the two structures. The membrane of the stressed ER triggers the recruitment of adaptor proteins such as FAM134B and ATL3 that have LC3-interacting regions, which can interact with nascent autophagosome membrane proteins such as ATG8, LC3 or GABARAP [38]. Then the autophagosome expands around the ER fragment that need to be removed and fuse with lysosomes wherein degradation of ER components takes place.

#### 3.2.3. Unfolded Protein Response

When the clearance capacity of misfolded or unfolded proteins is exceeded and they accumulate in the ER lumen, the unfolded protein response is triggered and orchestrates the enforcement of adaptive mechanisms to restore the homeostasis in protein synthesis.

**Figure 5:**
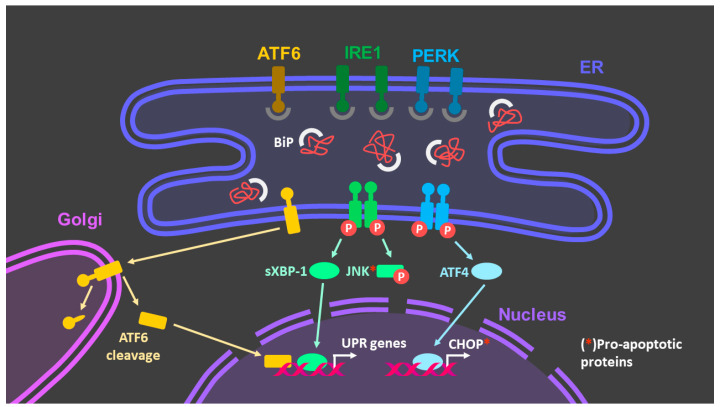
Activation of the unfolded protein response (UPR). The accumulation of misfolded proteins in the ER leads to the dissociation of BiP from the three ER-stress sensor PERK, IRE-1 and ATF6 and activates the coordinated unfolded protein response. ATF6 is translocated to the Golgi where it undergoes sequential cleavage and migrates to the nucleus. IRE-1 and PERK are activated by oligomerization and trans-phosphorylation. The IRE-1 endoribonuclease activity splices out an intron on XBP1 mRNA to generate XBP1s. IRE-1 also triggers the JNK pathway. PERK phosphorylates eIF2α (not shown) and enhance the translation of ATF4 which upregulates CHOP. The integrated signaling of this three sensor regulates mRNAs decay, attenuates translation, increases chaperon sintesis, upregulates the antioxidante response, and promotes ERAD and reticulophagy. Sustained hyperactivation leads to apoptosis.

The UPR is triggered by the combined action of three conserved ER transmembrane stress sensors: activating transcription factor 6α (ATF6), PKR-like ER kinase (PERK), and inositol-requiring enzyme 1α (IRE1α) [47] (Figure 5). Under homeostatic conditions, these three proteins are inactive due to association with BiP. However, as we have explained, BiP has a high affinity for misfolded proteins and is therefore in an equilibrium between binding to these sensor proteins and to misfolded proteins. If the misfolded proteins begin to accumulate in the ER-lumen, BiP will dissociate from the ER stress sensors triggering the downstream signaling cascade [48].

IRE1α. Dissociation of ATPase domain of BiP from IRE1α, or as it has been shown, direct binding of hydrophobic residues of misfolded proteins to IRE1α [49], triggers IRE1a dimerization and its subsequent auto-transphosphorylation, activating the endoribonuclease domain of IRE1α, which induces splicing of the mRNA that encodes unspliced XBP1 (XBP1u) to generate spliced XBP1 mRNA that encodes a potent transcriptional factor, spliced-XBP1 (XBP1s) [50]. XBP1s target genes involved in ER protein folding, protein translocation and secretion, as well as and increased degradation of misfolded proteins by the ERAD. In addition, IRE1α activity also induces regulated IRE1α-dependent mRNA decay (RIDD), which functions to reduce levels of mRNAs limiting newly synthesized proteins [51].

PERK. PERK activation by oligomerization and subsequent auto-transphosphorylation upon BiP dissociation leads to phosphorylation of eukaryotic translation initiation factor 2 subunit-α (eIF2α) which inhibits ribosome assembly and, therefore, reducing the overall initiation of mRNA translation giving the cell a temporary rest to manage ER stress [48]. Activating transcription factor 4 (ATF4) mRNA, escapes translation inhibition and is preferentially translated in the presence of eIF2α [29]. ATF4 translocates to the cell nucleus and induces the transcription of genes involved in amino acid biosynthesis, the antioxidative response, and reticulophagy [50].

ATF6α. After BiP dissociation from ATF6α, ATF6α is transported to the Golgi apparatus where it is cleaved by the Golgi proteases site-1 protease (S1P) and site-2 protease (S2P) to release an active cytosolic ATF6 fragment (ATF6p50) [52]. This fragment migrates to the nucleus and operates as a transcription factor that activates transcriptional programs that reinforce the ERAD pathway and modulates XBP1 mRNA levels [53].

However, when these attempts to restore ER homeostasis fail and ER stress-induced damage is irreversible, UPR signaling is activated to induce apoptosis, a process known as the terminal UPR.

PERK hyperactivation can upregulate the CHOP transcription factor, which inhibits expression of the gene encoding the B cell lymphoma 2 (BCL-2) protein. BCL-2 acts as an anti-apoptotic protein that hasten cell death. CHOP also upregulates growth arrest and DNA damage-inducible 34 (GADD34) that may induce the generation of reactive oxygen species (ROS). PERK hyperactivation also promotes the expression of pro-apoptotic BCL-2 members such as BIM. Sustained IRE1 hyperactivation promotes apoptosis signals through the activation of c-Jun NH2-terminal kinase (JNK). IRE1 could also be detrimental to the ER homestasis by degrading the mRNA of essential cellular folding machinery such as chaperones by RIDD. Altered calcium homeostasis owing to inositol-1,4,5- trisphosphate receptor (IP3R) activation secondary to IRE1 activation, in addition to ROS, may also contribute to the opening of the mitochondrial permeability transition pore (PTP), which promotes apoptosis.

### 3.3. ER-Stress and Aging

Maintenance of proteostasis deteriorates with aging, leading to a progressive increase in ER-stress [54]. With age expression and activity of many key enzymes of the adaptative mechanism to ER-stress decline compromising proper protein folding and driving this mechanism towards pro-apoptotic signaling. Critical chaperones are progressively oxidized in the ER lumen contributing to their functional deterioration [55]. Moreover, not only does their function deteriorate, several studies have shown that the expression levels of BiP, PERK, eIF2, ATF4, Gadd34, IRE1, and XBP1 decrease with age [56,57]. However, other studies have found contradictory results, suggesting that these age-related changes may be tissue dependent [56]. Moreover, adaptive responses to cellular stress are dynamic changes that respond to acute and chronic alterations that occur in tissues with age and whose response is not unique but varies according to the intensity and origin of the stimulus and can be considered a dose-dependent homeostatic response [58]. Consequently, it is not surprising that contradictory results have been found regarding the expression levels of these proteins.

In contrast to the reduction in expression and activity of many chaperones with age, CHOP and other pro-apoptotic proteins are elevated. In aged animals, prolonged ER stress leads to a more deleterious outcome compared to younger individuals which supports the idea that the aged animals are more sensitive to apoptosis. It has been reported that CHOP expression levels are elevated in aged animals both at baseline and after stress induction [59,60] and when compared to young, stressed animals, CHOP and caspase-12 expression was only elevated in aged animals [61]. However, CHOP is not only involved in apoptotis, it has also been described to play a role in metabolic and immunomodulatory responses [62,63,64], so it may be that increased CHOP activity in aged individuals is associated to inflammation rather than apoptosis [58]. Levels of activated phospho-JNK are also elevated during aging which could further shorten the threshold of apoptosis [65].

In many cases it is difficult to distinguish whether aging-associated changes in the UPR are a contributing cause or a consequence of aging, linked to senescent cells. However, some authors have shown that selective activation of specific UPR signaling pathways are capable of inducing a senescent state in cells and that blocking these pathways could partially reverse the senescent state [66,67,68,69].

Loss of chaperone function can lead to loss of the native soluble form of proteins and the formation of insoluble aggregates with cytotoxic consequences. Intracellular inclusion bodies formed by misfolded proteins have long been identified as possible inducers of many neurodegenerative diseases which is why ER-stress have been proposed as a potential inducer of many age-related neurodegenerative diseases such as Parkinson’s [70], Alzheimer’s [70], amyotrophic lateral sclerosis [70], and Huntington’s Disease [71]. The role of ER-stress and its therapeutic potential remains to be determined in many of these diseases and certainly deserves further study.

### 3.4. ER-Stress and AMD

The most frequent cause of ER-stress is the disturbance of the redox balance in the reticulum lumen [72]. This is especially relevant for the retina because it is one of the tissues with the highest metabolic rate and oxygen demands in the body and is therefore exposed to high levels of oxidative and photooxidative damage throughout life [73], in fact, oxidative stress play a central role in the development of AMD [74] and ER-stress has been proposed as a key pathogenic mechanism in AMD development because of its association with angiogenesis oxidative stress, and apoptosis [75].

The redox balance of the intracellular environment can have a major impact on proteostasis. The ER lumen has a more oxidizing redox potential compared to the cytosol in order to facilitate disulfide bond formation during protein folding [76]. The formation of disulfide bonds together with the degradation of misfolded proteins by ERAD are redox-dependent processes that lead to a net loss of reducing equivalents [77,78], this may have a great impact considering the large amount of proteins that are subjected to both processes during normal protein folding.

Glutathione (GSH) is the main redox buffer of the cells, including the ER, and allows native disulfide bonds to form [79]. During intensive disulfide bond formation GSH is consumed and cells respond to this threat by up regulating GSH biosynthesis, but ER stress can exceed this compensatory mechanism and lead to an increasingly reduced ER environment and the excess of ROS begins to accumulate [80]. Under these circumstances of excessive oxidative stress, the normal protein folding is altered, and illegitimate disulfide bond formation and stabilization of undesirable conformations become frequent, increasing the amount of proteins targeted for ERAD. However, before degradation disulfide bonds must be reduced, but the large consumption of reducing equivalent limits this step [81]. Consequently, ER stress can disrupt redox balance and trigger oxidative stress, and oxidative stress can impair chaperone function, induce the formation of aberrant disulfide bonds and impede protein degradation causing further ER stress [82].

In aged RPE, impaired proteasome activity is observed as well as increased oxidation of cellular proteins [83]. In these cells, oxidative stress inactivates the proteasome, promoting ER stress, and seems to be one of the mechanisms to explain the accumulation of ubiquitin conjugates upon oxidative stress [84]. Proteasomal inhibition can also increase lipofuscin formation [85], a hallmark of AMD pathogenesis [86]. Proteasomal over-inhibition results in the expression of hypoxia-inducible factor (HIF)-1α and NF-kappaB, factors that regulate the expression and secretion of proangiogenic factors such as VEGF [87].

ER stress in the neural retina has been shown to activate autophagy and lead to a secondary proteasome insufficiency and sensitize cells to apoptotic pathways [88]. Treating with selective phosphodiesterase-4 inhibitor to increase proteasome activity inhibits ER stress-related autophagy and attenuates retinal degeneration [89]. In contrast, when proteasome activity is inhibited in the RPE, there is an increase in autophagic activity and confers resistance to oxidative stress damage [90]. These findings indicate that regulating the flux of misfolded proteins targeted to autophagy or to the proteasome, may reduce proteotoxicity during ER stress and improve cell survival.

Regarding the UPR, a significantly reduced levels of XBP1s and an abnormal UPR response under ER-stress were observed in the retina of aged wild type mice. Furthermore, in KO mice that did not express XBP1 in the retina, a significant thinning of the retinal layers was observed [91]. It has been reported that XPB1 may play an essential role in the regulation of oxidative stress responses by controlling the expression of antioxidant genes such as catalase and superoxide dismutase 1 and 2 (SOD1, SOD2) in the RPE. In an RPE-specific conditional XBP1 KO mice the expression of these genes were significantly decreased while the ROS levels were markedly elevated in the outer retina and RPE and was associated by increased oxidative damage and cell death [92]. Interestingly, unspliced XBP1, but not spliced XBP1, overexpression re-establishes catalase expression in XBP1-deficient cells and ameliorates ROS formation [92]. Taken together, these findings demonstrate an essential role of XBP1 maintaining the redox homeostasis in the retina and modulating cell death. Furthermore, IRE1, XBP1-independent, ER stress-dependent mitochondrial apoptosis of human RPE cells via JNK pathway has also been reported [93].

Additionally, inhibition of PERK has been shown to aggravate misfolding and aggregation in the retina of mice genetically subjected to ER-stress, whereas treatment with eIF2α phosphatase inhibitors to increase PERK activation improves photoreceptor survival [94]. Concerning the ATF6 sensor, an ATF6-mediated stress response has been shown to be essential for color vision in humans since mutation in this genes results in achromatopsia [95].

The pathogenic role of inflammation in macular degeneration is an area of great interest [96] and the capacity of ER stress response to initiate and modulate inflammation is becoming increasingly relevant and could help to understand the role in this disease [97,98]. In fact, persistent activation of the UPR have been described to promote retinal degeneration via the UPR-induced pro-inflammatory cytokine IL-1β [99].

VEGF plays a key role in both angiogenesis and vascular permeability, which is why anti-VEGF therapy is currently the main therapeutic option [100]. Alterations caused by insufficient vascular supply such as hypoxia and nutrient deprivation are triggers for ER-stress [101]. Transcription factors from all three arms of the UPR have been shown to bind to the promoter region of VEGF a promote its transcription [102]. However, it appears that different stimuli may preferentially activate one arm of the UPR over others, for example hypoxia and glucose deprivation upregulates VEGF via the IRE1α pathway [103]. Pigment epithelium-derived factor (PEDF) is a potent angiogenic inhibitor produce by the RPE that counteract VEGF contributing to vascular quiescence while it also exerts several neuroprotective and antiapoptotic effects [104]. Interestingly, the unfolded protein response decreases PEDF expression in human RPE [105].

### 3.5. ER-Stress and Mitochondrial Dysfunction in AMD

Under conditions of moderate oxidative stress, induced by cigarette smoke extract, human retinal pigmented epithelium cells have been shown to trigger a robust response of the three UPR sensors that favors cell survival; however, mitochondrial function is prematurely impaired, decreasing ATP production and favoring ROS accumulation, adding stress to the ER, and inducing a retinal pigmented epithelial–mesenchymal transition, a change that is seen in early AMD [106]. However, under conditions of intense ER-stress, induced by tunicamycin, it has been observed that the RPE initiates a deleterious rather than protective UPR, and favors mitochondrial dysfunction [107]. In cultured rat retinal cells, induced mitochondrial dysfunction also induces ER-stress upon ATP depletion and increased ROS [43]. These data seem to indicate that modulation in the intensity of the UPR response may determine whether it acts to promote cell survival or apoptosis and points towards mitochondrial dysfunction as the origin of the cellular damage.

## 4. New Therapeutics for AMD

Currently, AMD therapeutic options are limited, anti-VEGF drugs are available to treat the wet form while for the dry form, administration of nutritional supplements is recommended but do not completely cure patients affected by AMD. Consequently, new therapies are needed beyond the well-known AMD-related molecules, lutein, zeaxanthin, vitamin D, and omega-3 acids [8,10,108,109]

As oxidative damage, impaired activity or function of the RPE, increased apoptosis, and chronic inflammation have been identified as keys processes in AMD, one of the potential new therapeutic options may be molecules that exhibit antioxidants properties. Therefore, we would like to review several molecules that have been shown to be potential candidates for the treatment or prevention of AMD in recent years as potential new therapeutic options.

### 4.1. Bile Acids

Bile acids, the major lipid components of bile, are synthetized in the liver from cholesterol as a precursor and stored in the gallbladder until the presence of fats and proteins following food intake in the stomach results in their release into the duodenum. During their intestinal transit, gut anaerobic microbiota flora produces the secondary bile acids by modification of the primary bile acids cholic acid (CA) and chenodeoxycholic acid (CDCA), the two primary bile acids in humans. The secondary bile acids deoxycholic acid (DCA) and lithocholic acid (LCA) are formed by dehydroxylation of CA and CDCA, respectively, while epimerization of hydroxyl groups of CDCA leads to the formation of ursodeoxycholic acid (UDCA). Both primary and secondary bile acids are normally conjugated to glycine or taurine in the liver. CA, DCA, and UDCA are conjugated with glycine to form glycocholic acid (GCA), glycodeoxycholic acid (GDCA), and glycoursodeoxycholic acid (GUDCA), respectively, and UDCA is conjugated with taurine to form tauroursodeoxycholic acid (TUDCA). In addition to increasing hydrophilicity, conjugation of bile acids with taurine or glycine increases solubility and limits passive diffusion across the cell membrane [110,111].

Traditionally bile acids were considered as digestive molecules, whose main function was to participate in the emulsification, absorption, and digestion of dietary fats and liposoluble vitamins, also playing a secondary role in the modulation of hepatic glucose metabolism and liver cell survival [110]. In fact, UDCA was originally approved by the US Food and Drug Administration (FDA) for the treatment of several forms of cholestatic syndromes due to its capacity to protect hepatocytes from hydrophobic bile acids and its choleretic effects [112]. However, recent findings have suggested the participation of bile acids in many different functions. T/UDCA, for example, has been shown to have not only an antiapoptotic effect by interfering with the mitochondrial pathway of cell death, inhibiting oxygen-radical production, and reducing endoplasmic reticulum stress and caspase activation, but also by its anti-inflammatory and antioxidant effects, as well as preservation of the blood–retinal barrier [113].

It is well known that the endoplasmic reticulum stress and apoptosis are important factors in many neurodegenerative diseases affecting the central nervous system and retina. In this sense, T/UDCA have proven to have a neuroprotective effect in several models of retinal disease, including photoreceptor degeneration (retinitis pigmentosa and light- induced retinal degeneration [114,115,116,117,118,119], Leber congenital amaurosis [120], and retinal detachment [121]), retinal ganglion cell degeneration (glaucoma and Leber hereditary optic neuropathy [122,123]) and diabetic retinopathy [124], at variable doses and routes of administration, and through different mechanisms of action [125].

As recent studies strongly correlate neurodegenerative disease to the state of the microbiome, authors have hypothesized that the effect that the alterations in the microbiota could have on circulating bile acids may induce or exacerbate neurodegenerative processes, and, in particular, retinal degeneration [125]. In this sense, two independent metabolome-wide association studies identified a set of metabolites that partially discriminated between neovascular AMD patients and controls, including altered plasma levels of glycine- and taurine-conjugated bile acids in neovascular AMD patients, suggesting that they may play a role in AMD pathophysiology [126,127]. As we have previously described, oxidative endoplasmic reticulum stress and apoptosis of RPE cells and photoreceptors are known to play an important role in AMD and the development of CNV, typical of exudative AMD. In recent years a number of in vitro assays have been carried out to assess the ability of bile acids to prevent features of AMD from developing or progressing. The majority of these studies have been performed on RPE and choroidal endothelial cells, the two cell types primarily involved in AMD pathophysiology.

In a study using ARPE-19 cells treated with H_2_O_2_, TUDCA protected against oxidative damage, attenuated cell death and significantly increased cell viability compared to cells exposed to H_2_O_2_ alone. Authors suggest that TUDCA decreased the generation of reactive oxygen species (ROS) and malondialdehyde (MDA), upregulated antioxidant gene expression, and increased the generation of glutathione (GSH). TUDCA also inhibited inflammation by decreasing the expression of proinflammatory cytokines and suppressed thapsigargin-induced ER stress in RPE cells, as demonstrated by decreasing the expression of CHOP and apoptosis [128]. In another study, also performed on ARPE-19 cells, TUDCA was able to reverses H2O2-induced phagocytosis impairment through the activation of Mer tyrosine kinase receptor (MerTK) and focal adhesion kinase (FAK) pathways [129].

In two independent studies, Warden et al. recently assessed the ability of glycine and taurine-conjugated bile acids at different concentrations, to inhibit features of AMD modeled in vitro. On the one hand they evaluated their capacity to protect human primary RPE cells tight junction integrity, assessed by ZO-1 immunofluorescence and transepithelial electrical resistance measurements, against paraquat-induced oxidative damage; and on the other hand, they tested their ability to inhibit VEGF-induced choroidal endothelial cells angiogenesis, evaluated through cell proliferation, cell migration, and tube formation assays in immortalized macaque choroidal endothelial cells [111]. With these experiments, authors found that GCA, GDCA, and GUDCA preserved RPE tight junctions’ function and structure under oxidative stress conditions. While none of the studied bile acids affected VEGF-induced choroidal endothelial cells’ proliferation, GCA and GUDCA, but not GDCA, significantly inhibited VEGF-induced cell migration and tubulogenesis [111].

With regards to their taurine-conjugated bile acids study, taurocholic acid (TCA) also proved to protect against oxidative stress-induced damage to RPE tight junction structure and function, probably due to the capacity of taurine to inhibit ROS; however, the exact mechanism is not completely clear [130]. In the choroidal endothelial cells angiogenesis assays, TCA inhibited VEGF-induced choroidal endothelial cell migration and tube formation, but showed no effect in VEGF-induced cell proliferation [130]. These results suggests that both glycine- and taurine-conjugated bile acids may be capable of stabilizing the RPE under severe oxidative stress, by protecting against the invasion of aberrant choroidal vessels into the subretinal space in neovascular AMD, and inhibiting crucial steps of choroidal angiogenesis.

Recent exudative AMD models have evaluated the effects of bile acids in laser-induced CNV. Woo et al. demonstrated that the intraperitoneal administration of T/UDCA, significantly suppressed laser-induced CNV formation in rats, probably due to its anti-inflammatory action, showing less fluorescein leakage and reduced CNV lesion sizes. VEGF showed significantly lower levels than controls, but only in the group of rats treated with TUDCA, suggesting that UDCA could have a different mechanism of action [131]. Moreover, Maharjan et al. recently develop an oral UDCA formulation that efficiently delivered UDCA to the eye tissues through the blood–retinal barrier. This study showed that UDCA formulation not only down-regulated VEGF expression and reduced CNV vascularity and size, but also promoted the functional recovery of the mice retinas according to electroretinography evaluation. Therefore, the authors suggest that UDCA oral formulation could be used as a supplement in cases of exudative AMD thanks to it being a safe and cost-effective way to deliver drug molecules to the eyes [132].

All of these findings suggest that bile acids would activate different signaling pathways in RPE and choroidal endothelial cells (in fact several receptors and transporters for bile acids have been identified in retinal cells). However, little is known about the role of endogenous circulating bile acids in the retina, and how bile acids specifically interact with these receptors or transporters [125]. A bile acid transporter called OATP1B3 has been found to be expressed in the retina and has been shown to prefer taurine- and glycine-conjugated bile acids over unconjugated bile acids [133,134]. Additionally, conjugated bile acids have been shown to activate other receptors like TGR5 and S1P2, suggesting that differences in hydrophobicity are relevant and can alter the effect of bile acids on endothelial cells [111].

Although there is increasing evidence in the literature to support the potential role of bile acids as protective agents against the progression of AMD, more studies are needed to understand the molecular mechanisms and signaling pathways through which these molecules protect RPE cells and inhibit angiogenesis in choroidal endothelial cells. Their benefit in clinical setting remains poorly investigated and, to date, there is no clinical study evaluating bile acids as a treatment for AMD, but further investigation regarding this therapeutic option seems promising.

### 4.2. Humanin

Humanin (HN) is a mitochondrial-derived peptide encoded in the mitochondrial genome by the 16S ribosomal RNA gene. Depending on whether the translation takes place in the cell cytoplasm or mitochondrion, a 24- or 21-amino acid peptide will be produced, both of them with demonstrated biological activity. HN is expressed endogenously by several cells and tissues in the body (brain, colon, testis, heart, kidney, skeletal muscle, and liver). Further, endogenous HN has been reported to be secreted from cells into the plasma and transported to targeted tissues that express HN receptors, where it could activate extracellular and intracellular signaling pathways [135]. In the extracellular pathway, HN triggers several downstream signaling cascades, such as JAK2-STAT3, P13-AKT, or extracellular signal-regulated kinase 1/2 (ERK1/2) after binding to the heterotrimeric HN receptor, while intracellularly, HN performs its functions through IGFBP-3, Bax, Bid, BidEL, and tBid [136].

Since its initial discovery over a decade ago, mounting evidence has linked HN to biological processes such as apoptosis and cell survival, and HN has shown cytoprotective, cardioprotective, and neuroprotective effects in many cell types in vivo and in vitro, playing a key role in the cellular stress response under different stressors including Aβ oligomers [137], tert-butyl hydroperoxide (tBH)-induced stress [138], and endoplasmic reticulum (ER)-stress [139]. Therefore, it has been evaluated as therapeutic target for different chronic diseases including Alzheimer’s disease, stroke, diabetes, myocardial ischemia and reperfusion, atherosclerosis, amyotrophic lateral sclerosis, and certain types of cancer [135,140].

More recently, the protective role of HN in the RPE has been investigated. In this sense, many studies have revealed that HN treatment can significantly protect RPE cells from oxidative and ER stress-induced cell death and exhibits activity against cell senescence [138].

Sreekumar et al. were the first to report that HN is expressed in the cytoplasmic and mitochondrial compartments of human nonpolarized and polarized RPE cells [35], and that exogenous HN is rapidly taken up by RPE cells where it is targeted to the mitochondria [138]. They also demonstrated that human RPE cells express HN trimeric receptor complex consisting of the cytokine receptor (WSX-1), the transmembrane glycoprotein 130 (gp130), and ciliary neurotrophic factor receptor (CNTFRα), which are essential for the extracellular action of HN. Sreekumar et al. described that HN plays a protective role against oxidative stress by enhancing mitochondrial function and biogenesis through the increase of mitochondrial DNA mass, mitochondrial number, and protein expression level of mitochondrial transcription factor, mtTFA, a key protein involved in mitochondrial biogenesis. Moreover, the authors reported that one of the extracellular mechanism through which HN performs its action is by activating the signal transducer and activator of transcription 3 (STAT3) by phosphorylation and inhibiting caspase-3 activation after binding to the receptor. Moreover, co-treatment with HN maintained transepithelial resistance of polarized RPE monolayers from oxidative stress-induced cell injury, suggesting that HN has a potential protective role in the normal RPE monolayer [138].

In line with these results, the same group later reported that HN has shown a protective effect against ER stress induced apoptosis in primary human RPE cells. HN pretreatment significantly decreased the number of apoptotic cells with all three ER stressors, tunicamycin (glycosylation inhibitor), brefeldin A (Golgi complex translocation inhibitor), and thapsigargin (calcium flux inhibitor), in a dose dependent manner. This beneficial effect was achieved through the attenuation of activated caspase 3 and ER stress specific caspase 4, restoration of depleted mitochondrial GSH, and suppression of ER stress induced mitochondrial superoxide production [139].

HN has also been linked to cell senescence. Circulating levels of HN have been shown to fluctuate with age in mice and humans, showing a decrease with age that suggests that HN might be an antiaging peptide whose decline could play a role in the pathogenesis of age-related diseases [138]. In a H2O2-induced human primary RPE senescence model, HN significantly reduced the classical markers of senescence such as senescence-associated β-Gal-positive cells, ApoJ transcripts, and p16INK4a expression [138]. As previously mentioned, Amyloid-β is a key component of drusen deposits and is also involved in senescence-related AMD pathogenesis. Administration of a HN inhibits amyloid-β-induced cell apoptosis in AMD RPE cells cybrids [137], by restoring amyloid-β-mediated decline in calcium homeostasis, suppressing amyloid-β-induced membrane fluidity changes and mitochondrial membrane potential, and decreasing intracellular ROS [141].

HN has also demonstrated a role in the treatment of the neovascularization that occurs in wet age-related macular degeneration. In a recent study, a more potent HN derivative, AGA-HN, exhibited the ability to reduce the expression of VEGF in ARPE-19 cells in addition to protecting cells against oxidative cell cytotoxicity, suggesting that HN could represent an alternative or complement to the current anti-VEGF therapies [142].

HN extensive functions in the ocular tissue include neuroprotection to the recently described chaperone-mediated autophagy. Numerous studies have demonstrated that both autophagy and heterophagy (phagocytosis of exogenous photoreceptor outer segments) are highly active in the RPE, and increasing evidence shows that the constant oxidative stress, can lead to decreased autophagy and heterophagy in the RPE cells and consequently AMD progression [143]. Therefore, it should be acknowledged that autophagy may represent an important therapeutic target in AMD, and in this sense, HN has demonstrated to directly activate chaperone-mediated autophagy (CMA) by increasing substrate binding and translocation into lysosomes [144].

The growing evidence of the cytoprotective effects of HN has led to the development of HN analogs through single amino acid substitutions, that exhibit specific agonistic or antagonistic properties, for the treatment of chronic diseases. Humanin G (HNG), which is produced by substituting serine with glycine at position 14, confers 1000-fold more cytoprotective activity than endogenous HN against neurotoxic insults, and is known to protect against cell death by preventing mitochondrial dysfunction [135]. In ARPE-19 AMD cybrids, exposure to HNG protected mitochondria, downregulated pro-apoptosis genes, and increased the protection against Aβ-induced damage, promoting increased cellular longevity. These results suggest that exogenous HNG acts via both intracellularly (binding to BAX protein, which prevents the release of cytochrome c, and consequently inhibiting apoptosis) and extracellularly (binding to gp130 receptor complex, inducing activation of JAK-STAT pathway and transcription of downstream genes) [137].

It is not surprising that HN has become an attractive therapeutic candidate for AMD. Intravitreal injections are the most effective way to deliver HN peptides to the retina. However, because of the poor stability and rapid clearance of small peptides such as HN, frequent dosing will be required to maintain therapeutic levels in the vitreous. In the past few decades, considerable advances have been made in ocular delivery systems [136]. Recently, Li et al. described two genetically-encoded fusions between HN and elastin-like polypeptides (ELP), HN-S96 and HN-V96. Both HN-ELPs successfully bind and activated human RPE cells and significantly protected RPE cells against oxidative stress induced cell death via STAT3 activation. No evidence of mitochondrial translocation of HN-ELP nanoparticles was observed, therefore, the authors hypothesized that HN-ELPs may work differently from HN free peptide, and STAT3 pathway appears to be a major pathway involved in HN-ELP mediated cellular protection [145]. Solanki et al. also described a HN drug delivery system of the more potent HN derivative, AGA-HNG, encapsulated in chitosan nanoparticles (NPs) using an ionic gelation method. They demonstrated that he chitosan NPs exhibited similar anti-VEGF properties and oxidative protection as the free protein in vitro using ARPE-19 cells, while exhibiting superior biocompatibility and drug release [142]. Despite these findings, more studies about HN ocular pharmacokinetics and pharmacodynamics will be necessary to clarify its clinical applications for long term use.

### 4.3. Resveratrol

Resveratrol (RSV) is a trans-3,4′,5-trihydroxystilbene, a major polyphenol produced in a small number of plant species that can be found in grapes, peanuts, and red wine [146]. RSV has been proven to modulate multiple targets in numerous pathologies such as coronary heart diseases, cancers, inflammatory, and age-related degenerative diseases [147]. RSV can act at multiple levels such as cellular signaling, enzymatic pathways, apoptosis, and gene expression [147]. Its antioxidant effect occur mainly via scavenging of ROS, while its benefit on inflammatory diseases is related to its ability to decrease the production of pro- inflammatory cytokines [148]. Moreover, RSV also contributes to age-related degenerative diseases thanks to its capacity to activate sirtuin-1 (SIRT-1) [149].

Due to its antioxidant properties, RSV could play a role in ocular tissue protection; in fact, several studies have demonstrated a protective effect of RSV against RPE cells oxidative stress, the primary cell target for AMD. King et al. have shown that RSV inhibited basal and H2O2-induced intracellular oxidation, protecting ARPE-19 cells from H2O2-induced cell death and hyperproliferation through the inhibition of mitogen-activated protein kinase (MAPK)/ERK 1/2 pathway [150]. Moreover, RSV significantly enhances cell viability and promotes cell growth, by modulating superoxide dismutase (SOD)/malondialdehyde MDA) activity and inhibits apoptosis by activating Bcl-2 expression and suppressing cleaved caspase 3 expressions in RPE cells exposed to H2O2 [151]. As mentioned above, RSV also acts as an efficient anti-inflammatory molecule and was found to protect RPE cells from sodium iodate, inhibiting ROS and interleukin (IL)-8 production on an atrophic AMD model [152]. A recent study also found that RSV reduced the UVA-induced death via suppressing the generation of intracellular H2O2, MAPK activation, and COX-2 expression [153]. In addition, RSV has demonstrated an antiaging effect in vitro, where it counteracted the detrimental effect on DNA methyltransferases (DNMT) and SIRT1 functions and restored nuclear element-1 (LINE-1) methylation [149].

Smoking is a significant environmental risk factor associated with neovascular AMD and geographic atrophy. Acrolein, the major toxicant in cigarette smoke, it is known to induce oxidative stress in human RPE cells and inhibit their phagocytic function. On a recent study, pretreatment with RSV demonstrated a protective effect against the damage caused by acrolein in human RPE R-50 cells [154]. Another cigarette smoke-derived toxic compound, hydroquinone (HQ) [155], also induces cytotoxicity, increases apoptosis and oxidative stress in RPE cells, and trigger VEGF production. An RSV and omega-3 fatty acids containing the product (Resvega^®^) was able to improve RPE cell viability, reduce ROS production and modulates inflammatory chemokines, IL-8, monocytic chemoattractant protein (MCP)-1, and IL-6 [156]. In another study, Neil et al. showed that cultured human RPE cells treated with the combination of RSV and HQ had significantly increased cell viability, improved mitochondrial function, upregulated antioxidant genes, activation of the ER stress response, and direct oxidant interaction [157].

One of the first changes observed during early AMD is the accumulation of lipofuscin in RPE, which is associated with the decline of autophagy during aging. In this sense, two independent studies demonstrated that RSV induced autophagy in ARPE-19 cells by modulating p62 and LC3 levels, along with increasing the number of autolysosomes [158,159]. RSV also exhibits a protective effects against N-retinyl-N-retinylidene ethanolamine (A2E), a major lipofuscin component, through attenuating A2E cytotoxic effect, strengthening the cell monolayer integrity, maintaining the intracellular redox balance, and preventing A2E-induced mitochondrial network fragmentation [160]. Moreover, on another study RSV and its metabolite, piceatannol, protected ARPE-19 cells from A2E and blue-light-induced photo-damage, increased cell viability, and reduced intracellular A2E accumulation in RPE cells [161].

With regards to CNV, both in vitro and in vivo studies, have provided evidence that RSV exerts anti-angiogenic effects on AMD. RSV significantly suppressed VEGF-A and VEGF-C secretion induced by inflammatory cytokines, TGF-β and hypoxia on human RPE cells, without affecting its basal secretion, critical for maintenance of healthy blood vessels in the choroid and retina [162]. In ARPE-19 cells, RSV significantly inhibited the expression and secretion of VEGF by inhibition of HIF [163]. RSV used at a concentration found in human plasma following oral supplementation protects ARPE-19 cells against the pro-angiogenic activity of oxysterols, and demonstrated a marked decrease in VEGF secretion [164]. Moreover, RSV caused inhibition of growth, migration, and tube formation of choroidal endothelial cells in vitro and suppressed VEGFR2 phosphorylation, both basal [165] and induced by VEGF [166]. In another in vitro study in ARPE-19 cells, red wine extract seemed to modulate the signaling pathway leading to VEGF production, but a decrease in the activation of the VEGF-A receptor, VEGF-R2, and MEK and ERK 1/2 pathways [167].

In mice laser-induced CNV models, orally administration of RSV significantly reduced blood vessel formation [163,165,168]. In addition, in vivo studies showed that RSV inhibited Akt/protein kinase B activity in choroidal endothelial cells and activated p53 [165], inhibited macrophage infiltration and inflammatory and angiogenic cytokines into the RPE-choroid complex, including VEGF, monocyte chemotactic protein-1 (MCP-1), and intercellular adhesion molecule-1 (ICAM-1) [168]. Intravitreal injection of RSV significantly inhibited FA leakage, CNV volume and area in laser-induced CNV [166]. Finally, in a laser-induced model of CNV, our group demonstrated that, when compared to untreated controls, mice treated with a modified AREDS II nutritional supplement containing resveratrol, alone and in combination with an intravitreal anti-VEGF, showed a decrease in VEGF and MMP-9 gene expression and protein activity levels, along with reductions in CNV leakage and lesion size, with no significant differences between the two groups [169]. 

In addition to all the scientific evidence, that has demonstrated the benefits of RSV against AMD in preclinical studies both in vitro and in vivo, some clinical trials have also been conducted in recent years. A clinical trial performed in octogenarians with AMD in the USA showed a bilateral anatomic restoration of retinal structure and improve visual function after a daily oral administration of an RSV based nutritional supplement called Longevinex^®^ over short [170] and long term [171]. Our group also performed a randomized, observer-blinded trial to evaluate the efficacy and safety of the original AREDS formulation with a product that adds RSV (Retilut^®^, laboratorios Thea, Barcelona, Spain) in patients with unilateral wet age-related macular degeneration, and demonstrated that RVS containing supplement was associated with significant reductions in the plasma levels of some inflammatory cytokines such as interferon-γ, IL-1β, IL-8, and tumor TNF-α, when compared with the control group that received the original AREDS formulation only, after one-year follow-up [172].

After all the beneficial properties of RSV discussed above it is still necessary to ensure that adequate concentrations penetrate the eye after oral administration and that it has no significant adverse effects. Many formulations have been developed to efficiently deliver RSV into the eye, including nanoparticle complexation and lipid-based encapsulation. In a cellular model of AMD with ARPE-19 cells, RSV was loaded in a poly(lactic-co-glycolic acid) (PLGA) polymeric nanoparticle, showing a potential cellular uptake and anti-angiogenic properties by inhibiting VEGF expression [173]. With regards to the safety of RVS, adverse effects are still unknown; however, no associated toxicity has been reported so far in in vitro or in vivo studies, and RSV has been well tolerated in clinical trials [147]. Moreover, Subramani et al. showed that RSV was able to reverse some adverse effects of bevacizumab treated observed on cultured ARPE-19 cells through both notch signaling activation and dephosphorylation of ERK 1/2 and MEK [174].

### 4.4. Coenzyme Q10

Coenzyme Q10 (CoQ10) is a two-part molecule composed of a benzoquinone ring, which has redox active sites, and a long polyisoprenoid lipid chain [175]. This lipid soluble essential molecule is endogenously synthesized de novo in the body, mainly in the mitochondria and secondarily in the endoplasmic reticulum–Golgi complex, and can be found in every cell in the human body, widely distributed in cellular membranes and bound to lipoproteins, in particular low-density lipoprotein (LDL) in blood plasma [176]. CoQ10 pool is found in two main forms: protein bound in the inner mitochondrial membrane (approximately 30%), where it mainly participates in oxidative phosphorylation; and not protein-bound, acting as a lipophilic antioxidant [177].

The lipophilic characteristic of CoQ10, together with its redox capacity allows this molecule to participate in multiple cellular pathways and functions. The best-known role of CoQ10 is its function as an essential electron carrier in the mitochondrial respiratory chain donating and accepting electrons on a continuous oxidation–reduction cycle, and also creating a proton (H+) gradient across the inner mitochondrial membrane by transporting H+ from the mitochondrial matrix to the intermembrane space to generate the energy required by the cell for its biochemical functions [178,179]. CoQ10 passes electrons generated from the reduction of fatty acids and glucose between complex I (NADH-ubiquinone oxidoreductase) or Complex II (succinate-ubiquinone oxidoreductase) and Complex III (succinate-cytochrome c oxidoreductase) [178,179]. Moreover, CoQ10 also accepts electrons from several enzymes as a result of different metabolic processes, like sulfide quinone oxidoreductase (SQOR), proline dehydrogenase 1 (PDH), coline dehydrogenase (CHDH), mitochondrial glycerol-3-phosphate dehydrogenase (G3PDH), dihydroorotate dehydrogenase (DHOH), electron transport flavoprotein dehydrogenase (ETFDH), and hydroxylproline dehydrogenase. All these processes generate fully reduced CoQH2 (ubiquinol), which is re-oxidized to CoQ10 (ubiquinone) by complex III [175]. Therefore, adequate amounts of CoQ10 are necessary for cellular respiration and adenosine triphosphate (ATP) production, due to its location in the mitochondrial inner membrane and its role in the electron transport chain during aerobic cellular respiration.

Due to its lipid solubility, its redox capacity, its participation in lipid peroxidation, and its widespread distribution, CoQ10 in its reduced form (CoQ10H2), is considered one of the most efficient antioxidants. Its antioxidant function efficiently protects lipids from harmful oxidative damage, but also DNA and proteins [176]. CoQ10 is able to prevent lipid peroxidation in most subcellular membranes including mitochondria, due to its complex interaction during the peroxidation process, both preventing the production, and directly eliminating lipid peroxyl radicals [175]. In addition, CoQH2 antioxidant activity is markedly synergistic with vitamin E, due to the fact that CoQ10 regenerates vitamin E α-tocopheroxyl radical back to its biologically active reduced form, which has the ability to scavenge lipid peroxyl radicals [180]. Moreover, CoQ10 has demonstrated to prevent DNA damage, by decreasing strand breaks and oxidative damage, and promoting DNA repair enzyme activity [181]. CoQ10 also acts as a H+ transporter across lysosomes membrane to maintain an acidic pH, which allows lysosomes to carry out their function in degradation of cellular debris and maintaining intracellular integrity [182]. It has been suggested that CoQ10 could participate in the regulation of the mitochondrial permeability transition pore (PTP) and uncoupling proteins (UCPs); however, controversial results have been reported regarding both functions and further investigations are needed to confirm them. Furthermore, recent data shows that CoQ10 could have an effect in the expression of genes involved in human cell signaling, metabolism, and transport [176].

Due to its role in the mitochondrial electron transport chain, protection against free radicals, and support of lysosomal function, CoQ10 helps maintain a normal cellular function, especially in tissues with a high metabolic activity, where great amounts of ROS are generated in relation with high oxygen consumption. The retina is the most metabolically active tissue in the body, with the highest consumption of energy per unit area of tissue and is particularly susceptible to oxidative stress and lipid peroxidation due to its high proportion of polyunsaturated fatty acids (PUFAs), and its exposure to visible light [177]. CoQ10 has been detected in both the choroid and retina, although its levels are relatively low in comparison to other oxygen-demanding tissues, but there is increasing evidence that CoQ10 protects retinal cells in vitro and in vivo [177], and patients with primary CoQ10 deficiency may have retinopathy as part of their syndrome, which suggests that CoQ10 may play an important role in the pathogenesis of retinal conditions [183]. Moreover, it has been reported that the concentration of CoQ10 in the retina is higher than that of carotenoids and vitamin E in that tissue, suggesting that CoQ10 may act as a powerful antioxidant in the eye [184].

CoQ10 synthesis in the human body is known to peak around the third decade of life and subsequently decreases with age. This decline in CoQ10 plasma levels might contribute in part to some of the manifestations of aging and the development of chronic diseases in the elderly [185]. Decreased levels of CoQ10 with age is postulated to occur as a result of the reduction in biosynthesis along with an increase in degradation attributed to age-related modification in lipid membranes, which can alter quinone behavior [186]. In the retina, levels of CoQ10 also declines with age (by approximately 40%), with two consequences: a decrease in antioxidant ability and a decrease in ATP synthesis, that would exacerbate the risk of retinal disease and macular degeneration [184]. In this sense, it has been demonstrated that patients with wet AMD have lower CoQ10 levels in the plasma and platelets in comparison with age-matched controls [187]. Since platelets show similarities with neuronal cells, decreased antioxidants levels might indicate that a similar condition is taking place in the retina, which can favor the onset of AMD, in particular in genetically susceptible individuals. These results suggest that there is some relationship between CoQ10 levels and AMD [187].

In an initial pilot study in a small group of patients with early AMD, a mixture containing CoQ10 (30 mg/day) with acetyl-L-carnitine (ALC) (500 mg/day), PUFAs (1320 mg/day), and vitamin E (30 mg/day) were demonstrated to improve visual functions, that remained relatively steady after 24 months, assessed by macular photostress test and automatic perimetry, in comparison with age- and sex-matched patients affected by early AMD treated with vitamin E only (30 mg/day) [188]. These results were later confirmed in a bigger randomized, double-blind, placebo-controlled clinical trial where the mean change in all three parameters of visual functions (visual field mean defect, visual acuity, and foveal sensitivity measured by perimetry) showed a significant improvement in the treated group after 12 month of follow-up, together with a decreased in the drusen-covered area in comparison with the placebo group [189]. These findings strongly suggested that an appropriate combination of compounds which affect mitochondrial lipid metabolism, may improve and subsequently stabilize visual functions, and it may also improve fundus alterations in patients affected by early AMD [189].

From the analysis of the results of these clinical studies, where AMD patients showed a benefit from the intake of CoQ10 combined with ALC, PUFAs, and Vitamin E [188,189], the authors suggest that the positive effect could be related with the mitochondriotropic capacity of CoQ10 and the other compounds. This means that they have a particular affinity to mitochondria together with the capability of improving mitochondrial metabolism and restoring its membrane structure and function, which subsequently may improve certain metabolic processes of the RPE/photoreceptor/Bruch’s membrane complex, involved in visual functions, and therefore prevent or attenuate early AMD. Furthermore, the addition of highly concentrated n–3 fatty acids enhances the CoQ10 mitochondrial content and its antioxidant capacity [189]. This mechanism of action is essentially different from the beneficial effects that have been described for other antioxidants, vitamins, and lutein. Moreover, they may have different indications, as mitochondriotropic compounds seem to be effective for treating early AMD, while antioxidants seem to be more effective for preventing late complications in patients at high risk of developing advanced AMD [10].

The fundamental role of CoQ10 in mitochondrial bioenergetics and its well-acknowledged antioxidant properties constitute the basis for its clinical applications. Due to this, CoQ10 supplementation has been beneficial in the treatment of vascular diseases, diabetes, and neurodegenerative disease. CoQ10 treatment is safe and most clinical trials have not reported significant adverse effects in humans that necessitated stopping therapy [176,190]. Furthermore, new formulations that increase CoQ10 absorption and tissue distribution have been developed [191]. A significant body of evidence supports the role of CoQ10 in promoting retinal health through the inhibition of ROS production and protection of neuroretinal cells from oxidative damage. Although further studies are required to learn more about the pathophysiology of AMD and the potential beneficial effects of CoQ10 in its treatment, the existing data goes in favor of this molecule as an alternative and/or complementary therapy for early AMD.

### 4.5. Melatonin

Melatonin (N-acetyl-5-methoxytryptamine) is a hormone with a clear daily rhythm and an acrophase at night, secreted into the bloodstream by the pineal gland of all mammals, including humans [192]. The production of melatonin has also been demonstrated in multiple extrapineal tissues, including the retina, where photoreceptors seem to be the main source, but recent evidence suggests that it can also be produced by the inner retina and RPE. In the retina, melatonin can act as a paracrine modulator and is responsible for several regulatory functions, both receptor (MT1 and MT2)- and non-receptor mediated [193].

Melatonin, secreted from photoreceptors at night, may activate melatonin receptors to regulate retinal circadian functions, including the cyclic photoreceptor outer segment disc shedding and phagocytosis, increase retinal sensitivity to light, and release retinal neurotransmitter [194]. In addition, melatonin may also play a role protecting cells from oxidative stress [195,196]. Inside the cells, melatonin has a preference location for the nucleus, cell membrane, and mitochondria, the sites with more ROS production, [197] where it can directly neutralize free radicals and reactive oxygen and nitrogen species or can bind to its membrane receptors to participate in multiple signaling pathways, which protect cells from oxidative stress damage and promote enzymes that metabolize oxidative stress [198]. In the mitochondria, melatonin can contribute to preserve mitochondrial homeostasis, protects mitochondrial ATP synthesis, counteracts oxidative mtDNA damage and restores the mitochondrial respiratory control system [195].

Based upon its unique properties listed above, as antioxidant and modulator of many important retinal functions, and the fact that the circadian rhythmicity of melatonin production is often disrupted in the elderly, melatonin has been proposed as a potent therapeutic option for a number of age-related ocular diseases, including AMD [192,196,199]. Furthermore, a decrease in melatonin synthesis has been described with age, and the amount of 6-sulfatoxymelatonin in nocturnal urine (a parameter for estimating peak circulating melatonin levels) was lower in AMD patients than in age-matched controls, which supports the role of melatonin in the pathogenesis of AMD [200].

The antioxidant effect of melatonin could be crucial for its protective role in AMD [194], and a number of studies have evaluated this effect in ocular cells and tissues in vitro [201,202,203]. Liang et al. reported that treatment with melatonin minimized cell death and mitochondrial damage in ARPE-19 cells that were exposed to H_2_O_2_; however, the dosage of melatonin in this study significantly exceeded physiological concentrations found in the bloodstream [201]. Later, Rosen et al. demonstrated that melatonin showed protective effects on ARPE-19 cells against H_2_O_2_, at both physiologic and pharmacological concentrations. In addition, they found that luzindole, a melatonin receptor antagonist, completely blocked melatonin’s protective effects at low concentrations of melatonin but not at high concentrations, which suggest that melatonin´s protective effect may depend on the activation of its membrane receptors at low concentrations, and through both direct antioxidant and indirect receptor activation effects at high concentrations [202]. Recently, Yan et al. found similar results, where melatonin reduced H_2_O_2_-induced apoptosis and mitochondrial dysfunction in RPE cells, and MT1 knockdown blocked such protective role, suggesting that melatonin exerts its protective role against oxidative stress via Melatonin-MT1 signaling in ARPE-19 cells [203].

Other authors have hypothesized that melatonin could exert additional functions through down-regulation of hTERT (catalytic subunit of telomerase) expression and stimulating telomerase activity, an enzyme implicated in maintaining the length of telomeres, to rebuild the telomeres in senescent RPE cells [197]. In addition, melatonin was shown to decrease the level of mRNA for AhR-dependent genes indirectly involved in antioxidant defense in the retina of premature aging OXYS rats, a reliable model of age-related macular degeneration in humans [204].

As explained above, impaired autophagy has been associated with AMD, mainly through the accumulation of lipofuscin and extracellular drusen and melatonin has shown to influence autophagy and apoptosis in several studies. Chang et al. found that melatonin treatment significantly decreased the apoptotic rate in H_2_O_2_-induced RPE cell damage, by reducing the Bax/Bcl-2 ratio and the expression levels of the apoptosis-associated proteins cytochrome c and caspases. Moreover, melatonin increased the autophagy effect through upregulating the expression of the autophagy-related proteins LC3-II and Beclin-1, and downregulating the expression of p62 [205].

As part of the search for clinical applications of melatonin in AMD a few ex vivo studies have been performed. In an experimental model of nonexudative AMD in mice induced by superior cervical ganglionectomy (SCG), melatonin administered as subcutaneous pellets, was capable to significantly preserve visual functions as well as RPE and outer retinal structure, and also prevented the increase of oxidative stress markers. These results were observed when the melatonin treatment was started both at 48 h and 4 weeks post-SCG, which means that melatonin could not only prevent, but also slow or reverse nonexudative-AMD progression [206]. On a photoreceptor degeneration mice model induced by an intraperitoneal administration of N-methyl-N-nitrosourea (MNU), melatonin was injected into the vitreous body, and was able to ameliorate effectively the MNU induced photoreceptor degeneration and also improved the visual signal transmission within inner retinal circuits by inhibiting apoptosis and mitigating oxidative stress [207].

Due to its amphiphilic feature, melatonin can cross the blood–retinal barrier, and can ameliorate nitric oxide (NO) and VEGF production, reducing vascular permeability in the retina of hypoxic rats, which may be crucial in preventing the development of CNV in exudative AMD [208]. On another study, melatonin significantly reduced the size and volume of CNV lesions, suppressed vascular leakage, and inhibited vascular proliferation by switching the macrophage/microglia polarization from M2 phenotype to M1 phenotype via inhibition of RhoA/ROCK signaling pathway in a laser-induced CNV model in mice [209].

We have also explained before the strong relationship between smoking and AMD. Bardak et al. reported that melatonin and memantine (MMT) showed a protective effects against ethylpyridine (a component of cigarette smoke) induced oxidative stress, apoptosis and mitochondrial dysfunction in ARPE-19 cells in vitro, through significantly decreasing ROS and VEGF levels, caspase-3 and -9 activities, as well as pro-caspase and poly(ADP-ribose) polymerase expression [210].

To date only one clinical study has explored the treatment of AMD with melatonin supplementation. Yi et al. performed a case control study with a follow-up of 6-to-24 months, where 100 patients with both dry and wet AMD were given 3 mg melatonin orally each night at bedtime. After at least 3 months of treatment, the authors found encouraging results, the majority of patients had a reduction in pathogenic macular changes with no significant side effects [211]. However, larger randomized studies are still required to confirm these preliminary promising results of melatonin supplementation on the progression of AMD.

To summarize, melatonin is involved in the modulation of many important retinal functions, as indicated above, including reduction of oxidative stress, inflammation, and apoptosis in the retina, restoration of inner blood–retina barrier integrity, and reduction of VEGF and NO levels. These findings suggest that melatonin, a very safe compound which lacks proven adverse effects even at high doses in humans, could become a promising therapy to prevent, slow down or even reverse AMD [212].

## 5. Conclusions

With the increased aging of the population, chronic and age related disease are becoming a challenge for health systems. Many of these conditions share common factors in their pathophysiology, including oxidative stress. More evidence is highlighting the importance of mitochondrial dysfunction in AMD. Although ER stress mechanisms are design to protect cells, chronic exposure to oxidative stress and inflammation in patients with AMD turns this protective response into a harmful process that induces apoptosis and enhances inflammation, mitochondrial dysfunction, and oxidative stress, creating a vicious loop that leads to AMD development or progression.

Current treatments for AMD focus mainly on the treatment of exudation from CNV in wet AMD, and although antioxidant formulations are used routinely in clinical practice, their benefits for patients are limited. In this review, we summarized some very promising results with new therapeutic options that are aimed to ameliorate ER stress and restore mitochondrial function, reducing oxidative stress and its related negative consequences in patients with AMD.
